# Gross N_2_O Production Process, Not Consumption, Determines the Temperature Sensitivity of Net N_2_O Emission in Arable Soil Subject to Different Long-Term Fertilization Practices

**DOI:** 10.3389/fmicb.2020.00745

**Published:** 2020-04-28

**Authors:** Chang Yin, Xiaoping Fan, Guochao Yan, Hao Chen, Mujun Ye, Liang Ni, Hongyun Peng, Wei Ran, Yuhua Zhao, Tingqiang Li, Steven A. Wakelin, Yongchao Liang

**Affiliations:** ^1^Key Laboratory of Environment Remediation and Ecological Health, Ministry of Education, College of Environmental & Resource Sciences, Zhejiang University, Hangzhou, China; ^2^College of Resources & Environmental Sciences, Nanjing Agricultural University, Nanjing, China; ^3^College of Life Sciences, Zhejiang University, Hangzhou, China; ^4^New Zealand Forest Research Institute Limited (Scion), Christchurch, New Zealand

**Keywords:** gross N_2_O consumption, temperature, *nosZ*II, C_2_H_2_ inhibitor method, fertilization

## Abstract

Chronic amendment of agricultural soil with synthetic nitrogen fertilization and/or livestock manure has been demonstrated to enhance the feedback intensity of net N_2_O emission to temperature variation (i.e., temperature sensitivity, TS). Yet few studies have explored the relevance of changes in underlying gross N_2_O production and consumption processes toward explaining this phenomenon, in particular for the latter. Furthermore, the microbe-based mechanisms associated with the variation of N_2_O consumption process remain largely unexplored. To address this knowledge gap, a temperature- (15, 25, and 35°C) and moisture-controlled (50% water holding capacity) microcosm incubation experiment was established using an arable soil subject to long-term addition of synthetic fertilizer (NPK), a mixture of synthetic fertilizer with livestock manure (MNPK), or with no fertilizer treatment (CT). Over the incubation time period, the C_2_H_2_ inhibition method was adopted to monitor reaction rates of gross N_2_O production and consumption; the population sizes and community structures of *nosZ*I- and *nosZ*II-N_2_O reducers were analyzed using quantitative PCR (Q-PCR) and terminal restriction fragment length polymorphism (T-RFLP). The results indicated that only NPK significantly increased the TS of net N_2_O emission, and gross N_2_O consumption process consistently occurred under all treatment combinations (temperature and fertilization) at each sampling time point. The responses of gross N_2_O production and consumption processes to temperature elevation exhibited fertilization- and sampling time-dependent pattern, and the higher net N_2_O production TS in the NPK treatment was underlain by its higher TS of gross production process and insensitivity of gross consumption process to temperature. The size and structure of *nosZ*II-N_2_O reducers, as well as the community structure of *nosZ*I-N_2_O reducers, were positively correlated with variation of gross N_2_O production and consumption rates across all fertilization regimes. *NosZ*II-N_2_O reducer abundance was less responsive to temperature change, and its community structure less susceptible to fertilization, as compared with *nosZ*I-N_2_O reducers. Overall, our results demonstrate that the TS of the gross N_2_O production process, not gross consumption, is the key step regulating the TS of net N_2_O production, and both *nosZ*I- and *nosZ*II-N_2_O clades are likely active N_2_O reducers in the tested soil.

## Introduction

The emission of N_2_O in the soil/atmosphere interface is a result of production and consumption (reduction) processes (Chapuis-Lardy et al., 2007; Schlesinger, 2013). Tilting N_2_O balance toward reduction before its release from agriculture soils is of considerable interest in light of rapid global climate change, because N_2_O owns approximately 300 times stronger warming potential per unit than CO_2_ and strongly depletes stratospheric ozone (Ravishankara et al., 2009). Consequently, a better understanding of N_2_O turnover in agricultural soils is indispensable for devising management practices that mitigate N_2_O emission while maintaining crop production (Richardson et al., 2009; Bakken and Frostegard, 2017). By definition, this necessitates process-based researches distinguishing production from consumption (Conen and Neftel, 2006; Bakken and Frostegard, 2017). However, in comparison with ever-increasing knowledge on N_2_O production, at present little is known about the ecological significance of N_2_O consumption process (reviewed by Conen and Neftel, 2006; Chapuis-Lardy et al., 2007). Moreover, the sensitivity of this process to the variation of environmental parameters subject to global climate change and its associated microbial basis are not completely clear (Conen and Neftel, 2006; Chapuis-Lardy et al., 2007; Bakken and Frostegard, 2017; Hallin et al., 2018).

It has been extensively demonstrated that microbial nitrification and denitrification pathways account for the majority of N_2_O production in agricultural soils (e.g., Zhu et al., 2013), and they are influenced by several edaphic factors such as moisture content, soil physical structure, and temperature, etc., in addition to substrate availability (i.e., nitrogenous sources and organic carbon) (Butterbach-Bahl et al., 2013). Among these, the role of temperature is particularly relevant in the context of global warming, given that it impacts not only current but also future N_2_O emissions via positive or negative feedback (Smith, 1997; Paustian et al., 2016). What is more, recent studies indicate that amendment with synthetic nitrogen-based fertilizers (SNF) and/or livestock manure (LM) enhances the feedback intensity of nitrification- and denitrification-derived N_2_O to temperature elevation in agricultural soils, thereby increasing the temperature sensitivity (TS) of overall net N_2_O emission (Smith, 1997; Cui et al., 2016; Song et al., 2018). Emerging works posit that this, to a large extent, can be attributable to the shifts in community traits (i.e., community structure and abundance) and increase in the activities of N_2_O-producing microorganisms as a consequence of increase in substrate availability (Cui et al., 2016; Song et al., 2018). On the other hand, amendment with inorganic and/or organic nitrogen fertilizers typically results in the differentiation of soil physicochemical properties and the N_2_O-reducer’s community assemblage (Hallin et al., 2009; Guo et al., 2010; Qu et al., 2014; Cui et al., 2016), thereby exerting strong influences on the N_2_O consumption process (Richardson et al., 2009; Liu et al., 2014; Qu et al., 2014; Blum et al., 2018). It is thus reasonable to predict that the divergence in N_2_O consumption process among different fertilization regimes may also assume an important role in governing the net N_2_O emission and final variation of N_2_O emission TS in agricultural soils. However, currently, microbial mechanisms that control the gross N_2_O consumption process which influence the TS of N_2_O emission in arable soils under different fertilization strategies remain largely unexplored.

It is well known that biogenic consumption for N_2_O is only conducted by microorganisms that possess the nitrous oxide reductase enzyme encoded by *nosZ*gene (Richardson et al., 2009). Traditionally, the canonical denitrifiers (i.e., *nosZ*I N_2_O reducers) which are capable of complete denitrification (i.e., reduced nitrate or nitrite to N_2_, with N_2_O as an intermediate) are thought to be the sole biogenic N_2_O sink (Richardson et al., 2009), while recent surveys reveal that the genetic capacity for N_2_O reduction is much greater than previously expected, and some microorganisms catalyze the reduction of N_2_O to N_2_ via an atypical N_2_O reductase, hereafter referred to as *nosZ*II-N_2_O reducers (Jones et al., 2013; Sanford et al., 2013). Following field researches predict a key role of this clade in N_2_O consumption in soil (e.g., Jones et al., 2014; Domeignoz-Horta et al., 2015, 2016, 2018), a suite of physiological and biochemical analyses, however, reported conflicting results concerning the capacities of *nosZ*I vs. *nosZ*II- N_2_O reducers in reducing N_2_O (e.g., Yoon et al., 2016; Conthe et al., 2018). For instance, Yoon et al. (2016) found that several typical isolates of *nosZ*II-N_2_O reducers owned significantly lower whole-cell half-saturation constants and higher specific biomass per mol of N_2_O reduced than those of *nosZ*I clades. On the contrary, the findings of Conthe et al. (2018) showed that *nosZ*I-N_2_O reducers had higher specific growth rate than *nosZ*II clade microorganisms under growth-limited conditions, thus pointing out a more active role of the former in N_2_O consumption. Regardless, increasing evidence indicates that there exists niche differentiation between these two types of N_2_O reducers, and the *nosZ*II-N_2_O reducer is more sensitive to environmental variations than *nosZ*I clade (reviewed by Hallin et al., 2018).

The presumably key role of *nosZ*II-N_2_O reducers in mediating soil N_2_O sink capacity and niches partitioning between the *nosZ*I- and *nosZ*II-N_2_O reducer are implicated in N_2_O emissions from arable soils (Hallin et al., 2009, 2018; Jones et al., 2014; Cui et al., 2016; Juhanson et al., 2017). This is exemplified by works demonstrating that higher *nosZ*II diversity was congruent with a lower potential N_2_O/(N_2_O + N_2_) ratio in the field (Domeignoz-Horta et al., 2015), and that the decreasing ratio of *nosZ*I to *nosZ*II abundance, as a result of varying land management practices, coincided with lowered emission rates of N_2_O at a land scale (Juhanson et al., 2017). Accordingly, the differentiation of soil physicochemical properties caused by chromic addition SNF and/or LM is expected to lead to more drastic change in *nosZ*II-N_2_O reducers’ community than their *nosZ*I counterpart, thereby inducing the variation of the N_2_O emission TS. However, this hypothesis has not yet been tested.

Therefore, the objective of this study is to investigate the role of gross N_2_O production vs. consumption processes in influencing the net N_2_O emission TS in soil subject to different fertilization regimes and identify the potential microbial ecological mechanisms underpinning the variation of gross N_2_O consumption among different fertilization regimes. To achieve this, an arable soil in which long-term fertilization of SNF was found to significantly enhance the TS of net N_2_O was revisited (Yin et al., 2017). For comparison, plots receiving both SNF and LM (i.e., MNPK treatment) were further included in the assessment. We established a temperature and moisture-controlled microcosm incubation experiment during which gross N_2_O production and consumption rates were measured using the C_2_H_2_ inhibition method, as described by Yang et al. (2011). In conjunction with molecular ecological analysis of the community structure and abundance of *nosZ*I- and *nosZ*II-N_2_O reducers, we focus on testing the following hypotheses: (i) the divergence of not only N_2_O production but also consumption process in TS among different fertilization regimes plays an important role in determining their variation in net N_2_O emission TS; and (ii) the variation of N_2_O consumption processes is tightly linked to the shift of the community traits of both N_2_O- reducer guilds, in particular, *nosZ*II clade.

## Materials and Methods

### Soils Sampling and Description

Soil samples were collected on June 2015, after wheat harvest, from the Jintan Long-term Fertilization Experiment Station (31°39′N, 119°28′E), which is located at the southwest of Jintan county, Jiangsu province, China. The details of the experimental site have been provided previously (Yin et al., 2017). Briefly, the soil type in the experimental sites is classified as Gleyic Stagnic Anthrosol according to FAO system and has been cultivated under rice-wheat rotation regimes since the establishment of the experiment. Soil samples were collected in each of the four replicates of control (CT), NPK, and MNPK treatments in this study. For CT treatment, no fertilizer has been applied since the establishment of the experiment. For the NPK treatment plots, N, P, and K fertilizers were annually applied at rates of 300 kg ha^–1^, 120 kg P_2_O_5_ ha^–1^, and 100 kg K_2_O ha^–1^ in the form of urea, triple superphosphate, and potassium sulfate, respectively. However, only half amounts of N, P, and K of the same fertilizer form as used in the NPK treatment, in combination with 6000 kg ha^–1^ composted pig manure, were added annually for the MNPK treated plots. During the sampling campaign, seven cores of 5 cm diameter (0–20 cm) were collected in each field-plot replicate (*n* = 4) for each treatment. The cores were mixed thoroughly to make a composite sample and then shipped to the laboratory immediately. Because the soil samples were collected after a heavy rain, the sampled soils were air-dried under the ambient temperature (∼25°C) for 3 days to lower soil moisture, and then sieved through a 2 mm mesh. The moisture content of the sieved soils was adjusted to 30% water holding capacity (WHC), and stored at 4°C until use (about 6 months).

The bulk soil physicochemical properties were determined for each replicate of all fertilization regimes (*n* = 4) before the storage as follows: soil moisture was determined gravimetrically by drying at 105°C for 24 h; soil pH was measured in a 1:2.5 soil to H_2_O slurry mixture (w/v); total nitrogen (TN) was measured by the Kjeldahl nitrogen method; soil organic matter (OM) was measured using dichromate oxidation; soil available phosphorus (Olsen P) was determined by molybdenum antimony blue colorimetry after extraction with 0.5 M NaHCO_3_. The WHC was determined by saturating a subsample of soil in a glass funnel with bottom loosely plugged with a piece of cotton to drain for 2 h before determination of the gravimetric soil moisture content by drying at 105°C for 24 h. Soil NH_4_^+^ and NO_3_^–^ were determined before the setup of and during the incubation of microcosms using Lachat flow-injection auto-analyzer (Lachat Instruments, Mequon, WI, United States) after the soil samples were extracted with 0.5 M K_2_SO_4_.

### Microcosm Study

Stored soil samples were weighed (18 g on an oven-dried weight basis) into 125 ml serum bottles, followed by adjusting soil moisture content to 50% WHC with deionized water. After pre-incubation at 25°C for 3 days to restore microbial activity, four bottles from each fertilization treatment were sacrificed for determination of the initial NH_4_^+^ and NO_3_^–^concentrations, as well as the community traits of *nosZ*I- and *nosZ*II-N_2_O reducers. The remaining 324 microcosms were loosely capped with butyl stoppers and evenly assigned to 15, 25, or 35°C-adjusted incubators for a further 30 days’ incubation. Among these, 36 microcosms (i.e., 3 fertilization treatments × 3 temperatures × 4 replicates) were fixed for measurement of net N_2_O production over the incubation and served as 0 Pa C_2_H_2_ control for the estimation of gross production and consumption rate on Days 3, 15, and 30. The net N_2_O production rates were measured on Days 1, 3, 5, 7, 10, 15, 20, 25, and 30 of incubation. Before measurement, all serum bottles were ventilated for 15 min to keep an identical incubation condition, and then tightly sealed with butyl stoppers. Headspace gas samples (10 ml) were taken with a gas-tight syringe after 24 h. After gas collection, all bottles were ventilated for another 15 min and loosely resealed. During incubation, all microcosms were regularly ventilated and supplemented with deionized water to maintain soil moisture content.

On Days 3, 15, and 30, gross N_2_O production and consumption rates were determined using the C_2_H_2_ inhibition method (Yang et al., 2011) with minor modifications. Briefly, three headspace treatments with four replicates (*n* = 4): 0 Pa C_2_H_2_ (control), 10 Pa C_2_H_2_ (nitrification inhibited), and 10 kPa C_2_H_2_ (nitrification + N_2_O consumption inhibited) were used. Correspondingly, two bottles of each replicate per fertilization treatment under the same incubation temperature were randomly selected and assigned to 10 Pa and 10 kPa C_2_H_2_ treatments, respectively. Overall, in addition to the initial 36 bottles fixed to monitor net N_2_O production, another 72 microcosms were used. After ventilation for 30 min, the serum bottles were tightly sealed and the headspace gas replaced with the same amount of either air-diluted C_2_H_2_ or pure C_2_H_2_ (v/v, 99.9%) to reach target concentrations of C_2_H_2_. The headspace gas from the 0 Pa C_2_H_2_ treatment was replaced with the same amount of air to mimic possible interference of gas exchange. After 24 h incubation the gas samples were collected, the soil samples were destructively collected from a selection of bottles that were not treated with C_2_H_2_. These soil samples were used for DNA extraction and determination of NH_4_^+^ and NO_3_^–^, respectively. Concentrations of N_2_O were measured using gas chromatography (HP7890A, Agilent Technologies, CA, United States) according to Yin et al. (2017). Because of the potential heterogeneity associated with low amount of soil, we selected a 24 h incubation time rather than shorter period to detect statistically meaningful differences among C_2_H_2_ treatments (Yang et al., 2011). Moreover, a preliminary study indicated that the N_2_O production was nearly linearly related to time over a 24-h incubation period (data not shown).

### DNA Extraction and T-RFLP Analysis

DNA was extracted from 500 mg of soil using a Fast DNA SPIN Kit for soil with a FastPrep-24 machine (Qbiogene, Canada), and then checked by electrophoresis in 1% (wt/vol) agarose. For amplification of *nosZ*I and *nosZ*II gene fragments, primer pairs nosZF/nosZR and noZIIF/nosZIIR were used (Throback et al., 2004; Jones et al., 2013), with a fluorescent label (FAM) attached to each forward primer. For all amplifications, the 25 μl PCR amplification mix contained 2.5 μl of 10 × TaKaRa PCR Taq buffer, 200 μM of dNTP mix, 0.4 μM of each primer, 2.5 U Taq polymerase, and 400 ng μl^–1^ BSA. Thermocycling conditions were described previously (Jones et al., 2013; Cui et al., 2016). For each sample, PCR products from three independent runs were pooled to minimize PCR artifacts, and the expected band was gel-purified using Tiangen gel extraction kit (Tiangen, China). For enzyme digestion, around 500 ng purified PCR products were digested with 2 U *Bsl*I (for *nosZ*I) and *Ava*II endonuclease (for *nosZ*II), respectively. The digestion products were purified by a Tiangen DNA purification kit (Tiangen, China). Fluorescently labeled T-RFs were separated and detected using an ABI 3130 capillary sequencer (Applied Biosystems, Foster City, CA, United States).

The T-RFs’ patterns were assessed with the Peak Scanner software (Applied Biosystems). Peaks with T-RFs < 50 bp were discarded to eliminate background noises caused by primer dimers. The raw output of T-RFLP data was submitted to T-REX online pipeline^1^. After filtering data noise, T-RFs were aligned with the clustering threshold (bin) of 2 bp. To create the final sample × species (OTU) dataset, only peaks that occurred in more than 2% of the total numbers of samples were included, and T-RFs’ peak heights were relativized within samples.

### Quantitative PCR

Quantitative PCR was conducted in triplicate in an ABI7900 system by using SYBR green as the detection system. The 15 μl amplification mix for *nosZ*I contained 0.3 μM each primer, 7.5 μl of SYBR^®^ Ex TaqTM PCR mix (Takara, Dalian, China), 0.3 μl 50 × ROX dye, and 2 μl 20-fold diluted DNA. The 15 μl amplification mix for *nosZ*II gene detection contained 1.5 μM each primer, 7.5 μl of iQ^TM^ SYBER^®^ Green PCR mix (Bio-Rad, United States), 0.3 μl 50 × ROX dye (Takara, Dalian, China), and 2 μl of 20-fold diluted DNA. The amplification protocols were described elsewhere (Jones et al., 2013; Cui et al., 2016). Three replicates of no-template (negative control) were included; these gave null or negligible values. Standard curves were obtained by serial dilution of purified monoclone plasmids containing *nosZ*I and *nosZ*II genes amplified from the soil sample. All *R*^2^ were >0.99, and the amplification efficiency was 87.0% for *nosZ*I, and 90.2% for *nosZ*II.

### Statistical Analysis

#### Estimating the Temperature Sensitivity

As previously reported (Smith, 1997), we adopted *Q*_10_ to assess the overall cumulative net N_2_O emission TS spanning from 15 to 35°C. *Q*_10_ is an index indicating the response intensity of the biochemical reaction to temperature increase by 10°C. To estimate overall *Q*_10_, the cumulative net N_2_O emissions under different temperatures (i.e., 15, 25, and 35°C) were fitted with van’t Hoff equation to calculate two parameters α and β, which are shown as follows:

$$R=\alpha \,e^{\beta \,t};$$

$$Q_{10}=e^{\beta \times 10}$$

where *R* is the cumulative net N_2_O emission, *t* is the incubation temperature, α and β are fitted parameters. The overall *Q*_10_ was calculated from the latter equation.

However, the vant’s Hoff equation failed to capture the shift patterns of the gross N_2_O production and consumption rates across the whole temperature ranges tested, giving poor goodness of fit; this arose from the fact that these processes did not predictably respond to temperature elevation (Table 1 and Figure 2). Therefore, we instead assessed their importance in net N_2_O emission TS via simple linear regression and aggregated boosted tree.

**TABLE 1:** Summary of the effects of fertilization, temperature, sampling time, and interactions on the gross N_2_O production and consumption rate as well as the gene copy numbers of *nosZ*I and *nosZ*II-N_2_O reducers.

**Item**	**d.f.**	***F*-value**			
		**Gross production**	**Gross consumption**	***nosZ*I**	***nosZ*II**
Time	2	352.37***	454.99***	177.52***	273.17***
Fertilization	2	14.05***	6.31**	55.98***	259.40***
Temperature	2	5.28**	3.88*	17.61***	0.68
Time: fertilization	4	2.05	0.70	14.13***	8.37***
Time: temperature	4	1.06	0.44	47.26***	10.46***
Fertilization: temperature	4	4.32**	3.63**	16.04***	10.21***
Time: fertilization: temperature	8	3.77***	2.12*	32.73***	2.02
REML log likelihood		–164.99	–154.57	77.51	72.38

d.f., degrees of freedom. * P < 0.05, ** P < 0.001, *** P < 0.0001.

#### Statistical Analysis of Gross N_2_O Production and Consumption Rates

Two methods were used to calculate the gross N_2_O production and consumption rates in this study. First, following Yang et al. (2011), we used a simple arithmetic operation to estimate both process rates. The equations were shown as follows:

(1) $$R_{c\,o\,n}=R_{10\,k\,P\,a}-R_{10\,p\,a}$$

(2) $$R_{p\,r\,o}=R_{10\,k\,P\,a}-R_{10\,p\,a}+R_{0\,P\,a}$$

where, *R*_pro_ and *R*_con_ are gross production and consumption rate, respectively; *R*_0 Pa_, *R*_10 Pa_, and *R*_10 kPa_ are net N_2_O production rate under 0 Pa, 10 Pa, and 10 kPa C_2_H_2_ treatments of the corresponding replicate of each combination of temperature and fertilization treatment, respectively. The standard error of either process is propagated from the corresponding terms in the equations. A linear mixed model was then fitted for the analysis of gross production and consumption rates, with original fertilization block as a random effect and fertilization regimes, temperature, measuring time, as well as their interactions as fixed covariates.

Second, in the sense that the gross production rate is essentially derived from gross consumption rate via the method mentioned above, the standard errors of both processes are thus highly correlated. To minimize the influence of this correlation and test the robustness of statistical analysis mentioned above, the bootstrap method in combination with Monte Carlo simulation was further adopted to analyze the process rates of interest. Details of the procedure were described in Supplementary Material and related results were presented in Supplementary Figure S3 and Supplementary Table S3.

#### Statistical Analysis for Other Parameters

One-way ANOVA was used to evaluate the effect of temperature on net N_2_O production rates, NH_4_^+^ and NO_3_^–^ concentrations at each sampling time point within fertilization regimes. Tukey’s HSD pairwise comparison was used as a *post hoc* test for differences between temperature treatments. Two-way ANOVA was used to assess the effect of fertilization and temperature on cumulative net N_2_O production. Similar to gross N_2_O production and consumption rates, we adopted top-down strategies to build linear mixed effect models for the analyses of the abundances of *nosZ*I- and *nosZ*II-N_2_O reducers at Days 3, 15, and 30. The best-fitting model was selected via evaluation of Akaike Information Criterion (AIC) and log likelihood test. All data were tested for assumptions of normality and homogeneity of variance, log_e_- or log_10_-transformation was used to meet these assumptions when necessary. These analyses were conducted with “*nlmeU*” package in the R statistical environment (R Core Team, 2014).

For community structure analysis, the Bray–Curtis dissimilarity matrix was calculated for each clade based on the relativized T-RFLP data. Non-metric multidimensional scaling (NMDS) analysis was used to visualize distances among samples in two-dimensional ordination space. Permutation multivariate analysis of variance (PERMANOVA) was chosen to assess the effect of fertilization, temperature, and sampling time on the community structure of each functional guild based on 10,000 permutations. These were analyzed with “*vegan*” package (Oksanen et al., 2007). Linear regression was used to explore the relationship between process rates of interest, and the correlation of population size with process rates. The mantel test was used to assess the correlation between the community structures of functional guilds with the net N_2_O production, gross N_2_O production, and N_2_O consumption within fertilization regimes. The aggregated boosted tree was adopted to evaluate the relative importance of underlying processes, substrates availability, and community traits of functional guilds in predicting the net N_2_O emission, according to De’ath (2007). All calculations and analyses were conducted under R 3.4.2 statistical environment (R Core Team, 2014).

## Results

### Basic Soil Properties

Basic soil physicochemical properties were determined before the storage of samples (Supplementary Table S1). The results indicated that both NPK and MNPK treatments significantly declined soil pH, with the higher effect size observed for the former treatment; only MNPK treatment significantly increased the content of Olsen P, even though the Olsen P was about 3.5 times higher in NPK compared with CT. No significant difference was observed among treatments concerning TN, OM, and WHC, but these parameters showed a trend to increase following the order of CT < NPK < MNPK.

### The Net N_2_O Production Rates and *Q*_10_

**FIGURE 1:**
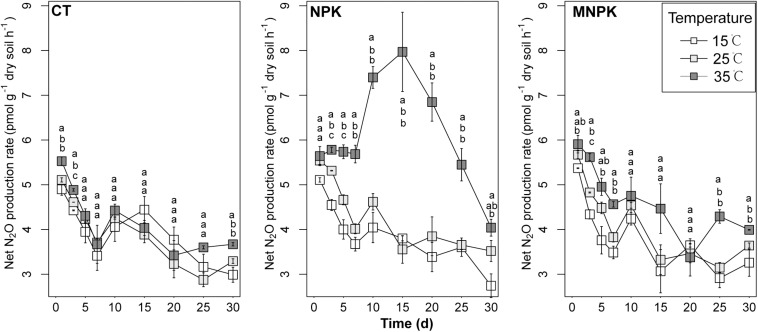
The net N_2_O production rates during 30 days’ incubation. The means ± 1SE (*n* = 4) are shown. Pairwise comparisons were conducted to assess the effect of temperature on net N_2_O production rate at a given day within fertilization regimes; significance level was *P* < 0.05. Different letters indicated significant differences among temperature treatments at a given sample time.

The net N_2_O production rate was monitored throughout 30 days’ incubation (Figure 1). With the exception of the NPK at 35°C where its rate held stable over the initial 7 days, the net N_2_O production rates of all other combinations of fertilization and temperature declined between Days 0 and 7, and the net N_2_O production rates of the most of samples incubated at 35°C were significantly higher than those at 15°C and/or 25°C during this period. Afterward, net N_2_O production increased to varying degrees depending on the combination of temperature and fertilization, and only rates of the NPK were significantly higher at 35°C than at 15 and 25°C between Days 10 and 20. At the later stage of incubation (i.e., 20–30 days), the net N_2_O production rates in NPK treated soils showed a trend of decline, while those of CT and MNPK did not exhibit any consistent trends across temperature treatments. At Day 30, the net N_2_O emission rates were generally significantly higher under 35°C than under other temperatures across fertilization treatments.

Two-way ANOVA analysis of cumulative net N_2_O emission indicated that higher emission occurred in samples incubated at 35°C than those at either 25°C or 15°C (*P* < 0.001) (Supplementary Figure S1), and NPK treatment had higher cumulative net N_2_O production than other fertilization treatments (*P* < 0.001). The calculation of TS showed that NPK had significantly higher *Q*_10_ than CT (*P* = 0.005) and MNPK (*P* = 0.006), and no significant difference was observed between the latter two treatments (*P* = 0.983) (Supplementary Table S2).

### The Gross Production and Consumption Rate of N_2_O at Days 3, 15, and 30

Pairwise comparison of net N_2_O production rates between C_2_H_2_ treatments revealed significantly higher net N_2_O production rate under 10 kPa C_2_H_2_ treatment as compared to 0 Pa and 10 Pa C_2_H_2_ treatments, suggesting a validity of C_2_H_2_ inhibition method (Supplementary Figure S2). While net N_2_O production rates of the majority of 0 Pa C_2_H_2_ treated soils were not significantly different from those of 10 Pa C_2_H_2_ treatment at 15 and 25°C, implying minor contribution of autotrophic nitrification to N_2_O production under these temperature regimes. In contrast, a considerable amount of N_2_O production derived from autotrophic nitrification in NPK treatment under 35°C at Day 15.

**FIGURE 2:**
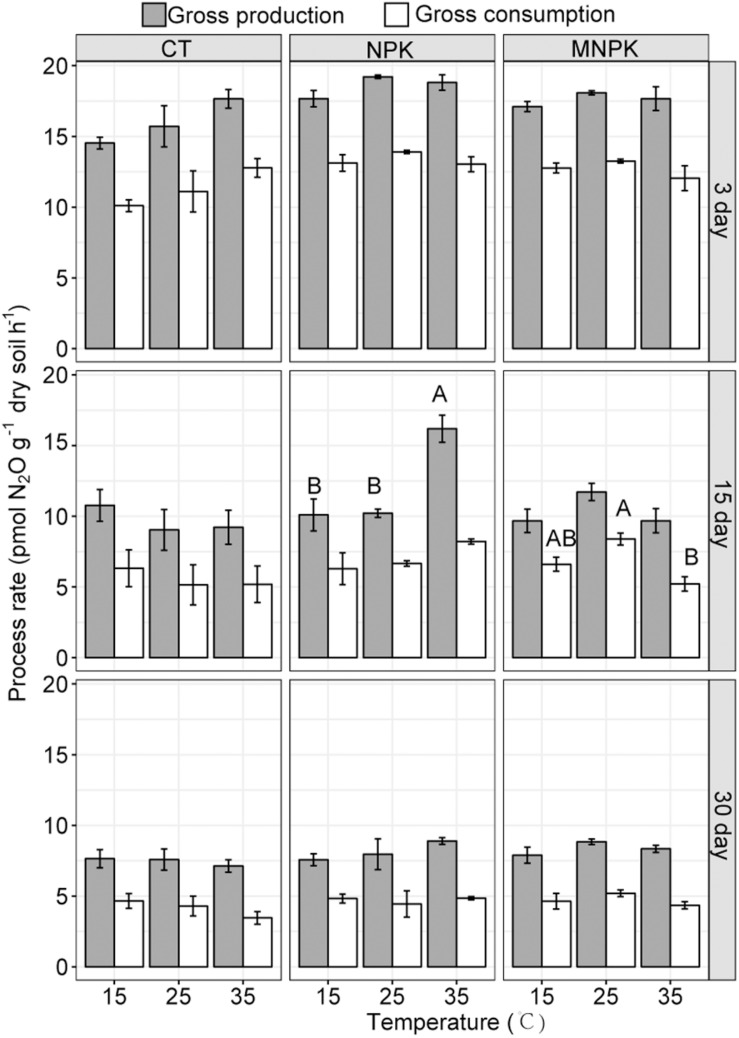
The gross N_2_O production and consumption rates calculated via simple arithmetic operation. Shown were means ± 1SE (*n* = 4). Different letters indicate significant difference among temperature regimes at a given sampling time.

Linear mixed effect models for gross N_2_O production and consumption rates estimated via simple arithmetic operation showed that both of them were significantly influenced by fertilization regimes, temperature, sampling time, the interaction between fertilization and temperature, and interactions among all factors (Table 1 and Figure 2). These results were further confirmed by the bootstrapped calculation and Monte Carlo simulation (Supplementary Table S3 and Supplementary Figure S3), and bootstrapped calculation of net N_2_O production rates gave similar results to parametric pairwise comparison among temperatures within fertilization regimes (Figure 1 and Supplementary Figure S3C). Specifically, both processes’ rates declined along with the incubation and were enhanced by NPK and MNPK treatment, with higher process rates being generally observed in the former treatment (Table 1, Figure 2, and Supplementary Figure S3). The effect of interaction of all factors on both processes arose from the following facts: both gross N_2_O consumption and production in CT treatment were enhanced by temperature elevation at Day 3, but the gross consumption declined with temperature at Day 30, and neither of processes showed an obvious trend to temperature elevation at Day 15 (Figure 2 and Supplementary Figures S3A,B); the gross N_2_O production and consumption rate of MNPK treatment peaked at 25°C across all measuring time points (Figure 2 and Supplementary Figures S3A,B); for NPK treatment, its gross production rate exhibited a trend of increase with temperature at Days 15 and 30, but gross N_2_O consumption rate was little affected by temperature treatments at Days 15 and 30 (Figure 2 and Supplementary Figure S3B).

### The NH_4_^+^ and NO_3_^–^ Concentrations at Days 0, 3, 15, and 30

The NH_4_^+^ and NO_3_^–^ concentrations were determined at Days 0, 3, 15, and 30 (Supplementary Figure S4). At Day 0, the NH_4_^+^ concentration of NPK averaged 13.05 μg g^–1^, being about 4 and 11 times higher than that of MNPK and CT, respectively (*F*_2_,_9_ = 556.14, *P* < 0.001), while no significant difference was observed between the latter two treatments (*P* > 0.05). After incubation under different temperatures, except in those samples from CT under 15°C as well as from NPK under 25 and 35°C, the concentration of ammonium generally decreased in all other samples and reached a relatively stable state in all treatments but NPK at Day 15. After 30 days’ incubation, the concentration of NH_4_^+^ in CT, NPK, and MNPK declined by 80, 24, and 36%, respectively, across temperature treatments, and no significant difference was observed among temperature treatments for MNPK (*F*_2_,_9_ = 3.23, *P* = 0.088), while significantly lower ammonium was observed for NPK under higher incubation temperature (*F*_2_,_9_ = 290.03, *P* < 0.001). However, the highest ammonium concentration in CT was found at 25°C (*F*_2_,_9_ = 6.28, *P* = 0.012).

By contrast, the concentration of NO_3_^–^ in NPK was found to be identical to that in MNPK at the starting of incubation, and was significantly higher than that in CT (*F*_2_,_9_ = 16.77, *P* < 0.001) (Supplementary Figure S4). Except for a sudden decline in NPK and MNPK under 35°C at Day 15, the concentration of NO_3_^–^ tended to increase throughout the whole incubation. The accumulation of NO_3_^–^ concentration was significantly enhanced by temperature across fertilization treatments (Supplementary Figure S4). The net increase of NO_3_^–^ concentrations was 68, 101, and 90 μg g^–1^ for CT, NPK, and MNPK across temperature regimes, respectively. Hence, approximately 99, 90, and 98% of NO_3_^–^ concentration buildup did not derive from the turnover of initial ammonium by autotrophic nitrification, thus implying a strong activity of heterotrophic nitrification or organic matter ammonification coupled with autotrophic nitrification.

### Community Structure of *nosZ*I and *nosZ*II N_2_O Reducers

**FIGURE 3:**
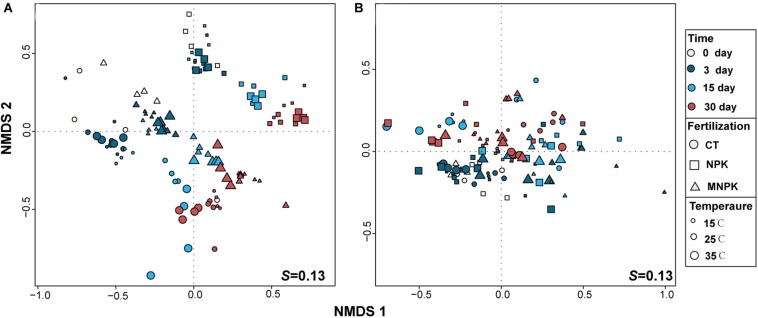
Non-metric multidimensional scaling (NMDS) analysis of T-RFLP profiles of *nosZ*I- **(A)** and *nosZ*II-N_2_O reducers **(B)**. The temperatures are scaled to the size of symbols, different colors represent sampling time, and the shape of symbols corresponds to different the fertilization regimes.

Non-metric multidimensional scaling was used to project the T-RFLP data of *nosZ*I and *nosZ*II-N_2_O reducers onto two-dimensional ordination space (Figure 3). A more regular distribution pattern of samples was observed for *nosZ*I-N_2_O reducers, in which three clusters corresponding to each fertilization regime were clearly detectable and samples of NPK were separated far away from MNPK and CT, indicating an overarching effect of fertilization on the community structure and stronger effect of NPK (Figure 3A). Meanwhile, distribution patterns corresponding to sampling time and temperature were discernable for *nosZ*I-N_2_O reducers depending on fertilization treatments (Figure 3A). These observations were all supported by formal testing by PERMANOVA, and significant interactions among combinations of each factor were also found (Table 2).

**TABLE 2:** Permutation multivariate analysis of variance (PERMANOVA) test for effects of sample time, temperature, and fertilization treatment on the community structures (genotypes) of *nosZ*I- and *nosZ*II-N_2_O reducers.

**Source of variation**	***F***	***nosZ*I**	***nosZ*II**
Time	*F*_3_,_90_	72.24***	37.23***
Fertilization	*F*_2_,_90_	269.50***	1.52
Temperature	*F*_2_,_90_	6.78***	18.22***
Time: fertilization	*F*_6_,_90_	5.85***	6.35***
Time: temperature	*F*_4_,_90_	9.16***	9.14***
Fertilization: temperature	*F*_4_,_90_	2.70**	14.16***
Time: fertilization: temperature	*F*_8_,_90_	3.08***	8.10***

** P < 0.001, *** P < 0.0001.

In contrast, the NMDS plot of *nosZ*II-N_2_O reducers could only be primarily split into two clusters along the vertical axis which matched the initial (i.e., Days 0 and 3) vs. later stage of incubation (Days15 and 30), suggesting a dominant effect of sampling time (Figure 3B and Table 2). The distribution patterns corresponding to fertilization regimes and temperature were unclear, while PERMANOVA indicated a significant effect of temperature but not fertilization (Table 2). PERMANOVA analysis also indicated that the community structure of *nosZ*II-N_2_O reducers was significantly influenced by the interactions of the combinations of each tested factor (Table 2).

### Abundance of *nosZ*I and *nosZ*II-N_2_O Reducers

**FIGURE 4:**
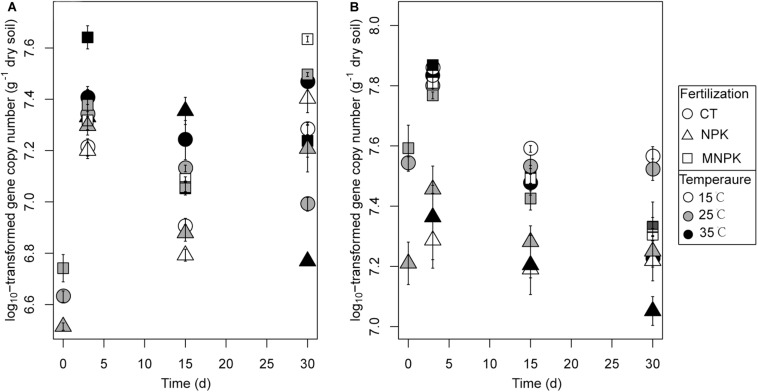
The copy numbers of *nosZ*I **(A)** and *nosZ*II **(B)** gene in soils following different fertilization treatments and incubated temperatures. Samples were taken at Days 0, 3, 15, and 30. Mean values ± 1SE (*n* = 4) are shown.

Using functional genes as biomarkers, we adopted q-PCR to determine the abundance of *nosZ*I- and *nosZ*II-N_2_O reducers throughout the soil incubations (Figure 4). At Day 0, the abundances of both guilds were significantly lower in NPK than in CT. During incubation, the abundance of *nosZ*I-N_2_O reducers was significantly influenced by all factors and their interactions (Table 1). The sampling time was the primary factor, which was reflected in the findings that *nosZ*I clade declined at Day 15 followed by an increase at Day 30 (Figure 4A). The effect of fertilization was due to that *nosZ*I-N_2_O reducers were lower in NPK than in CT or MNPK (Figure 4A). The effect of temperature was highly dependent on sampling time (Table 1), i.e., at Days 3 and 15 the abundance of *nosZ*I-N_2_O reducers showed a trend of increase with temperature but declined at Day 30 (Figure 4A).

The abundance of *nosZ*II-N_2_O reducers was strongly related to sampling time and fertilization regime (Table 1); this arose because of a continuous decline in abundance across all fertilization and temperature regimes after Day 3 and much lower abundance in NPK than in other treatments throughout the incubation (Figure 4B). In contrast, no temperature effect was found (*P* = 0.51), but the best-fitting linear model indicated significant interactions between temperature and either factor (Table 1).

### Correlation Analysis

**FIGURE 5:**
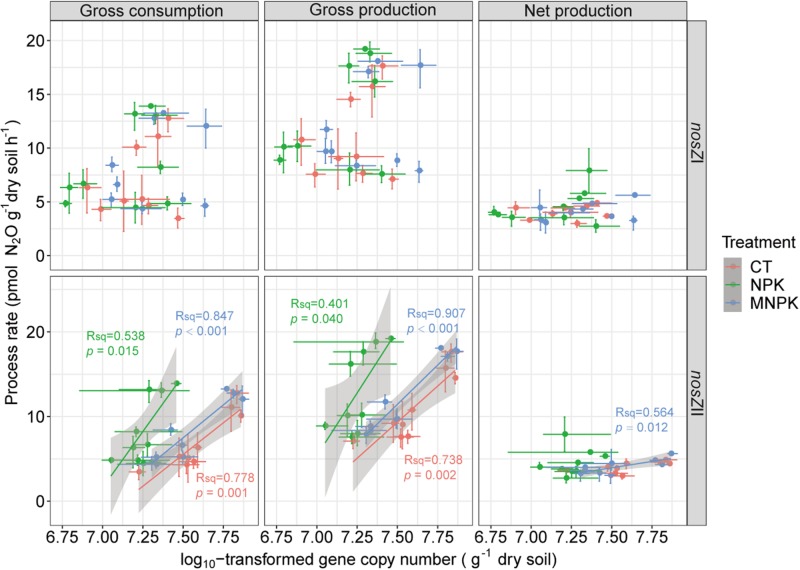
Linear regression analyses between related process rates including gross N_2_O consumption, gross N_2_O production, and net N_2_O production, with the abundances of *nosZ*I- and *nosZ*II-N_2_O reducers. The dots represent means and bars represent the 95% CIs.

Correlation analysis indicated that gross N_2_O production rate was highly correlated with gross N_2_O consumption rate within each fertilization treatment (*r* = 0.98, *P* < 0.001 for CT; *r* = 0.97, *P* < 0.001 for NPK; *r* = 0.98, *P* < 0.001 for MNPK) (Supplementary Figure S5). The gross N_2_O production rate of each treatment was also positively correlated with their own net production rates (*r* = 0.79, *P* = 0.002 for CT; *r* = 0.59, *P* = 0.046 for NPK; *r* = 0.71, *P* = 0.021 for MNPK). However, only the gross N_2_O consumption rate of CT was significantly correlated with its net N_2_O production rate (*r* = 0.83, *P* = 0.006). No significant correlations were established between the abundance of *nosZ*I-N_2_O reducers and all tested process rates (Figure 5); while the abundance of *nosZ*II-N_2_O reducers was significantly and positively correlated with gross N_2_O production and consumption rates across all fertilization treatments (Figure 5). Only the abundance of *nosZ*II-N_2_O reducers in MNPK was significantly and positively correlated with net N_2_O production rate (*r* = 0.75, *P* = 0.012). The mantel test indicated that the community structures of both functional guilds were significantly and positively correlated with gross N_2_O production and consumption rates within each fertilization regime (*P* < 0.001) (Table 3). Except in NPK treatment, the community structures of *nosZ*I- and *nosZ*II-N_2_O reducers in CT and MNPK were also significantly and positively correlated with net N_2_O production (*P* < 0.009).

**TABLE 3:** Mantel test between the community structures of *nosZ*I-N_2_O and *nosZ*II-N_2_O reducers with gross N_2_O production, gross N_2_O consumption, and net N_2_O production rate.

**Treatment**	**Item**	***nosZ*I-N_2_O reducers**			***nosZ*II-N_2_O reducers**		
		**CT**	**NPK**	**MNPK**	**CT**	**NPK**	**MNPK**
Gross production	*r*	0.537***	0.279***	0.695***	0.350***	0.353***	0.409***
Gross consumption	*r*	0.480***	0.373***	0.711***	0.348***	0.502***	0.371***
Net production	*r*	0.490***	−0.128	0.216***	0.216***	0.036	0.199***

*** P < 0.0001.

The relative importance of major measured parameters in predicting net N_2_O emission was evaluated by aggregated boosted tree (Supplementary Figure S6). The results indicated that the major part of variation of net N_2_O emission could be predicted by gross production, followed by nitrate; together they accounted for up to 75% variation. In contrast, gross consumption process could only capture 4.7% variation.

## Discussion

Amendment with SNF and/or LM could increase the *Q*_10_ of net N_2_O emission in arable soils, which was reported elsewhere (Smith, 1997; Cui et al., 2016; Yin et al., 2017; Song et al., 2018), and further confirmed in this study. However, in contrast to Cui et al. (2016), our results indicated that MNPK treatment had little influence on cumulative net N_2_O emission *Q*_10_. Given similar NO_3_^–^ concentration in NPK and MNPK treatment at the beginning of incubation in the tested soil, this phenomenon thus likely stemmed from much lower NH_4_^+^ concentration in MNPK, which might have severely restricted nitrifiers’ activity at the highest incubation temperature. Therefore, our results reaffirmed the importance of substrate availability in governing N_2_O emission TS in arable soils (Blagodatskaya et al., 2014; Song et al., 2018). However, instead of further dissecting mechanisms associated with the substrate-driven responses of N_2_O-producing microorganisms, here we focus on assessing the relevance of underlying gross N_2_O production and consumption processes to engendering this phenomenon, which has been rarely explored yet.

### Gross N_2_O Consumption Process Is an Integral Part of N_2_O Emission Under Aerobic Condition

In this study, the C_2_H_2_ inhibition was adopted to discriminate gross N_2_O production and consumption processes because of its simplicity and affordability (Groffman et al., 2006), and several reports revealed that it could give comparable estimates of either process rate to more sophisticated tools such as ^15^N isotope tracing (e.g., Yang et al., 2011; Zhu et al., 2013). However, it should be noted that this method would lead to varying underestimation of N_2_O production from denitrification depending on soil physicochemical properties including nutrient contents and moisture condition (Qin et al., 2013; Yuan et al., 2019), thus we readily concede that the results presented here might serve as conservative estimates of gross N_2_O production and consumption rate in the tested soil, and cannot be treated as exact representatives of field condition, albeit the low incubation moisture content and high organic matter of the tested soil might, to a large extent, alleviate this bias (Qin et al., 2013). Regardless, our results suggest that an appreciable amount of N_2_O consumption occurred under aerobic incubation condition (i.e., 50% WHC in this study). This is in line with previous reports in arable soils via multiple measurement approaches (e.g., Mathieu et al., 2006; Chapuis-Lardy et al., 2007; Yang et al., 2011; Wen et al., 2017; Yuan et al., 2019). Furthermore, the estimated gross consumption/production ratios [i.e., N_2_/(N_2_O + N_2_)] ranged from 0.48 to 0.75 with a mean of 0.62 across fertilization treatments. This overlapped with the value obtained by Yang et al. (2011) in a field measurement (0.19–0.83) and that of Mathieu et al. (2006) in a field survey (0.06–0.85), but was lower than that of Müller et al. (2014) (0.96–0.99) by calculating via ^15^N tracing analysis and that of Yuan et al. (2019) (0.93–0.97) by direct quantification N_2_. Overall, our results, together with others (Mathieu et al., 2006; Chapuis-Lardy et al., 2007; Yang et al., 2011; Müller et al., 2014; Wen et al., 2017; Yuan et al., 2019), underscore that gross N_2_O consumption is an integral part of N_2_O emission in arable soils even under aerobic conditions, and might occur in the anaerobic microsites of soils incubated.

### Gross Production Process Determines the Temperature Sensitivity of Net N_2_O Emission

Partitioning gross N_2_O production and consumption enables us to test the hypothesis that the variations of both processes are responsible for the divergence in net N_2_O production TS among different fertilization treatments. Our rationale for this hypothesis is that, both denitrification and nitrification are temperature-sensitive N_2_O-producing processes, and their responses to temperature are generally promoted by the increase in substrate availability due to either the addition of exogenous nitrogen source or the acceleration of intrinsic nitrogen transformation (Braker et al., 2010; Cui et al., 2016; Song et al., 2018). Meanwhile, gross N_2_O consumption process is theoretically less sensitive to temperature change in soils with lower pH (Richardson et al., 2009; Liu et al., 2014; Qu et al., 2014; Blum et al., 2018), as the activity of N_2_O reductase is highly susceptible to change in pH and will be impaired by lowering soil pH as a consequence of chronic amendment with SNF (Liu et al., 2014; Qu et al., 2014; Blum et al., 2018). Thus, it is expected that NPK treatment would show higher N_2_O emission TS than other treatments. In confirmation of this, we found that the gross N_2_O production rate in NPK treatment was the highest, and responded more positively to an increase in temperature than in CT and MNPK treatments, while its gross N_2_O consumption rate was little influenced by temperature.

Furthermore, our results revealed that the response patterns of underlying gross N_2_O production and consumption process under different fertilization regimes to temperature varied with the sampling time points. For instance, the gross N_2_O consumption rate of CT showed an opposite response to temperature at the initial vs. later stage of incubation, while the optimum reaction temperature for gross N_2_O consumption and production of MNPK seemed to be near 25°C across all measuring time points. By contrast, the gross consumption of NPK treatment was little affected by the imposed temperature at most of measuring time points, while its gross production rate exhibited a sheer increase with temperature at Days 15 and 30, resulting in an abrupt net N_2_O emission peak at Day 15 under 35°C. These response patterns of gross production and consumption to temperature led to their varying correlations with net N_2_O emission rates: the gross production correlated significantly with net N_2_O emission rates across all fertilization treatments, while a significant correlation between gross N_2_O consumption rate and net N_2_O emission was only established in CT treatment. Taken together, our findings underscored an overarching role of gross N_2_O production in regulating N_2_O emission TS in the tested soil, thus echoing the emerging viewpoint that the variation of net N_2_O emission is primarily driven by the gross production rather than the consumption process (Yang and Silver, 2016; Wen et al., 2017).

### Possible Microbial Ecological Mechanisms Underpinning the Variation of Gross N_2_O Consumption

A natural question arises as to which functional guild, the *nosZ*I-N_2_O and/or *nosZ*II-N_2_O reducer, is linked to the variation of N_2_O consumption process. Our results revealed that the *nosZ*II-N_2_O reducer might play an important role in mediating N_2_O consumption process under our incubation condition, as both its community structure and population size were significantly and positively correlated with gross N_2_O consumption rate across all fertilization treatments. This partly verifies our second hypothesis. Similarly, Jones et al. (2014) found that both the abundance and diversity of *nosZ*II-N_2_O reducers were positively correlated with the variation of N_2_O sink capacity across multiple soil types. Moreover, Domeignoz-Horta et al. (2016) reported that inoculation of high but not low concentration of *Dyadobacter fermentans*, a *nosZ*II-type N_2_O-reducing strain, could significantly reduce potential N_2_O production in more than 1/3 of the tested soils without changing potential denitrification (i.e., increased potential N_2_O consumption). Thus, our results add further evidence to the notion that *nosZ*II-N_2_O reducers could act as a N_2_O sink in soil (reviewed by Hallin et al., 2018).

Furthermore, in support of our second hypothesis, the gross N_2_O consumption rates significantly and positively correlated with the *nosZ*I-N_2_O reducer’s community structure across all fertilization regimes as well, albeit no significant correlation was established between its abundance and gross N_2_O consumption rate. The involvement of *nosZ*I-N_2_O reducers in N_2_O consumption varied among fertilization strategies, as higher correlation coefficients of *nosZ*I community with gross consumption rates were found in CK and MNPK than in NPK. Thus, it appeared that *nosZ*I clade played a more important role in N_2_O consumption in former two treatments. The notion that *nosZ*I-N_2_O reducers assume a role in N_2_O reduction is not novel (Richardson et al., 2009; Bakken and Frostegard, 2017; Conthe et al., 2018). For instance, Schulz et al. (2017) found that the field-scale low N_2_O emission rates in an organic agricultural soil were associated with the spatial-temporal distribution of *nosZ*I-N_2_O reducers’ abundance. Besides, a growing body of studies indicated that many typical *nosZ*I-type strains and enrichments, e.g., *Pseudomonas stutzeri*, can conduct N_2_O reduction under both aerobic and anoxic conditions (Miyahara et al., 2010; Park et al., 2017; Conthe et al., 2018). However, sequencing-based analysis of the targeted functional genes was not conducted here, thus the potential active lineages of *nosZ*I-reducers in the tested soil cannot be inferred, and further research using phylogenetic analysis is needed.

The correlation pattern between *nosZ*I- and *nosZ*II-N_2_O reducers with processes rates of interest is essentially underpinned by their varying responses to fertilization and temperature. Specifically, in line with others (Hallin et al., 2009; Cui et al., 2016), we confirmed that NPK treatment could significantly decrease the abundance of *nosZ*I-N_2_O-reducers compared with MNPK. Moreover, our results added that *nosZ*II-N_2_O reducers were more susceptible to NPK treatment than *nosZ*I clade in terms of population size (Figure 5). As proposed by Hallin et al. (2009), the decline of soil pH was likely the dominant factor responsible for the decrease in the abundance of either guild, since none of the measured physicochemical properties but pH significantly differed among all the tested fertilization treatments. Meanwhile, the finding that there is differential sensitivity to pH between *nosZ*I and *nosZ*II-N_2_O reducers corresponds well with the work of Jones et al. (2014) who demonstrated that *nosZ*II-N_2_O reducers were more sensitive to pH relative to *nosZ*I clade microorganisms.

Intriguingly, compared with *nosZ*I clade, the abundance of *nosZ*II-N_2_O reducers was little, if any, affected by temperature and their community structure was less susceptible to fertilization. The high sensitivity of the *nosZ*I-N_2_O reducers’ community structure to temperature variation has been reported previously (Wu et al., 2018), and shift in its community structure following different fertilization treatments is also documented by others (e.g., Hallin et al., 2009; Cui et al., 2016). The contrasting responses between *nosZ*I vs. nosZII-N_2_O reducers to fertilization and temperature might imply their differential preferences for temperature and variation of soil physicochemical properties as a result of different fertilizations, thus likely reflecting a niche differentiation as proposed by previous reports (Jones et al., 2014; Domeignoz-Horta et al., 2015, 2016, 2018; Hallin et al., 2018). However, in contrast to others (Domeignoz-Horta et al., 2015, 2016, 2018), our results suggested that the *nosZ*I-N_2_O reducers are more sensitive to the change in environmental factors, especially temperature, than their counterparts (*nosZ*II-N_2_O reducers).

### Caveat and Outlook

Interpreting the correlation analysis results into the causality between the activity of N_2_O-reducers and consumption rates, however, should be treated with caution, as the relationship between functional genes and/or transcript abundances with corresponding processes rate is not always as clear as reported here, and inconsistent results have been frequently reported (summarized by Rocca et al., 2014). Besides, counterintuitively, the community traits of both *nosZ*I- and *nosZ*II-N_2_O reducers were positively correlated with the gross production rates and net N_2_O production rates as well. This could be, on the one hand, attributable to the tight correlation between gross production and consumption process rates that might otherwise indicate a synchronized regulation of these processes under our incubation condition; on the other hand, we cannot exclude the possibility that some of *nosZ*I- and *nosZ*II-N_2_O reducers have the propensity to produce N_2_O, considering that conducting the N_2_O reduction step provides *nosZ*I-N_2_O reducers less energy gain (Hallin et al., 2018) and over half of known *nosZ*II-N_2_O reducers hold the genetic potential to produce N_2_O (Graf et al., 2014; Jones et al., 2014). Finally, the depletion of substrates as a result of the long-term storage of soil samples might lead to the underestimate of TSs of different fertilization regimes; however, our previous research with different preincubation procedure revealed similar net N_2_O emission patterns under CT and NPK, thus highlighting that the phenomena we observed here is reproducible (Yin et al., 2017). Overall, to establish a “true causality” between the activity of N_2_O-reducers and consumption rates, future work to take advantage of mRNA-based and/or stable isotope probing (SIP) methods is warranted. This is particularly true for N_2_O consumption process, given that its regulation of enzyme expression is notoriously complex (Richardson et al., 2009; Liu et al., 2014).

## Conclusion

Our results confirmed that chronic amendment with SNF significantly increased the *Q*_10_ of net N_2_O emission in arable soil. The higher net N_2_O emission TS in SNF treated soil was linked to its higher TS of gross N_2_O production and insensitivity of gross consumption process to temperature, which might, respectively, stem from high substrate availability and the retardation of gross N_2_O consumption by the decrease in soil pH. Furthermore, the response of gross N_2_O production and consumption to temperature increase both exhibited fertilization- and time-dependent pattern, highlighting a high degree complexity of regulation of these processes in arable soil. Analysis of N_2_O-reducers’ community traits indicated that *nosZ*I-N_2_O reducers were more sensitive to changes in soil physicochemical properties and temperature than *nosZ*II-taxa, and both guilds were likely active N_2_O reducers in our tested soil. However, interpreting the correlation between the community traits of N_2_O reducers and process rates of interest into causality should be treated with caution. To test our findings and pinpoint the ‘true’ active N_2_O reducers, works to take advantage of non-intervening tools such as ^15^N_2_O-pool dilution model in combination with mRNA-based and/or stable isotope probing (SIP) methods in diverse soil types are warranted. Altogether, our findings provide a new dimension to the mechanistic understanding of the variation of net N_2_O production TS in arable soil under different fertilization regimes.

## Data Availability Statement

The datasets generated for this study are available on request to the corresponding author.

## Author Contributions

CY and YL designed the research and wrote the manuscript with the contribution from SW, YZ, and TL. CY, XF, LN, and HP performed the data analysis and interpreted data. CY, GY, HC, and MY performed the experimental analysis. WR provided the experimental materials and methods. YL conceived the experimental design and supervised all aspects of experimentation, data analysis, and manuscript preparation.

## Conflict of Interest

The authors declare that the research was conducted in the absence of any commercial or financial relationships that could be construed as a potential conflict of interest.

## Footnotes

1 http://trex.biohpc.org/
