# Supplementary material for: Gross N2O Production Process, Not Consumption, Determines the Temperature Sensitivity of Net N2O Emission in Arable Soil Subject to Different Long-Term Fertilization Practices
Source: Front Microbiol. 2020 Apr 28;11:745. doi: 10.3389/fmicb.2020.00745 (PMC7198778; doi:10.3389/fmicb.2020.00745)
Supplement: Supplementary file 1 [file Data_Sheet_1.docx]

Supplementary Materials

1. **Supplementary methods**

Taking advantage of the fact that neither of best-fitting models for gross N2O production or consumption rate (calculated via simple arithmetic operation) retained a random effect of block, bootstrapping was then used (with 10000 iterations) to get robust estimates of the means and 95% confidence intervals (CIs) of these process rates; meanwhile the net N_2_O production rates were also calculated by this method for comparison (Davidson and Hinkley, 1997). The bootstrapped datasets for gross production and consumption rates were used to run Montel Carlo simulation. Although the majority of bootstrap data followed the normal distribution (Fig. S3), we still determined the related skew parameters (location, shape and scale) with “*sn.mple*” function in “*sn*” package (Azzalini, 2019). These parameters were used to simulate a new treatment-temperature-time-specific dataset for both processes’ rates with the ‘*rsn*’ function in “*sn*’ package. The number of new data points drawn from every distribution corresponding to each combination of temperature and fertilization at each measuring time were enlarged to 10 observations. Therefore, these new datasets assume difference among fertilization-temperature-time-specific distributions with regard to the effect of temperature, fertilization and measuring time, this is different from Averil et al. (2014). Consequently, these procedures lead us more likely to get positive statistical results but at high risk of type II error. The simulated dataset was then analyzed via general linear model with fertilization regime, temperature, measuring time and their interactions as fixed effect. The aforementioned procedure was repeated 10000 times, and the number of events that the fixed effects were significant at the *P* < 0.05 level was summarized and tabulated. We defined the number of events that the specific fixed effect was significant at *P* < 0.05 smaller than 500 in 10000 times simulation as low probability to occur (i.e. *P* < 0.05).

**Reference**

Averill, C., Turner, B.L., Finzi, A.C. (2014). Mycorrhiza-mediated competition between plants and decomposers drives soil carbon storage. *Nature* 505, 543-545. doi: 10.1038/nature12901.

Azzalini, A. (2019). The R package '*sn*': The skew-normal and related distributions such as the skew-t (version 1.5-4). URL <http://azzalini.stat.unipd.it/SN>.

Davison, A.C., Hinkley, D.V. (1997). Bootstrap methods and their applications. Cambridge University Press, Cambridge. ISBN 0-521-57391-2.

1. **Supplementary Tables and Figures**

**2.1 Supplementary Tables**

**Supplementary Table 1.** Soil physicochemical properties of different fertilization regimes before incubation. Shown are means ± SE (n=4).

| Treatment | pH | TN  (g kg^-1^) | OM  (g kg^-1^) | Olsen P  (mg kg^-1^) | WHC  (%) |
| --- | --- | --- | --- | --- | --- |
| CT | 6.77±0.04a | 1.70±0.04a | 14.34±0.73a | 7.14±1.24a | 59.06±1.43a |
| NPK | 5.54±0.08c | 1.87±0.06a | 18.80±1.45a | 25.25±3.90ab | 61.98±1.08a |
| MNPK | 6.24±0.23b | 2.02±0.15a | 20.77±1.20a | 36.13±7.17b | 62.95±2.81a |

**Supplementary Table 2.** The Q_10_ of cumulative net N_2_O emission under different fertilization treatments. The values are means ± SE (n = 4).

| Treatment | Q_10(15-35℃)_ |  |
| --- | --- | --- |
| CT | 1.09±0.03b |  |
| NPK | 1.28±0.04a |  |
| MNPK | 1.10±0.01b |  |

**Supplementary Table3.** The results of Monte Carlo simulation (10000 iterations). We defined the number of events with *p* < 0.05 smaller than 500 in 10000 times simulation as low probability to occur (i.e. *p* < 0.05), therefore, our simulation indicated all factors and their combinations exerted significant influence on gross production and consumption rates. Details on simulation see supplemental materials and methods.

| Source of variation | Number of events with *p* < 0.05 in 10000 times simulation | |
| --- | --- | --- |
|  | Gross production | Gross consumption |
| Fertilization | 10000 | 10000 |
| Temperature | 10000 | 9400 |
| Time | 10000 | 10000 |
| Fertilization:Temperature | 10000 | 10000 |
| Fertilization:Time | 10000 | 9999 |
| Temperature:Time | 10000 | 9963 |
| Fertilization:Temperature:Time | 10000 | 10000 |

**2.2 Supplementary Figures**

**Supplementary Figure 1.** The cumulative net N_2_O production during 30 days’ incubation. The cumulative net N_2_O productions were calculated using integration of the net N_2_O production rate over all sampling times. Two-way ANOVA was used to assess the effect of temperature, fertilization, and their interaction on cumulative net N_2_O emission.

**

**


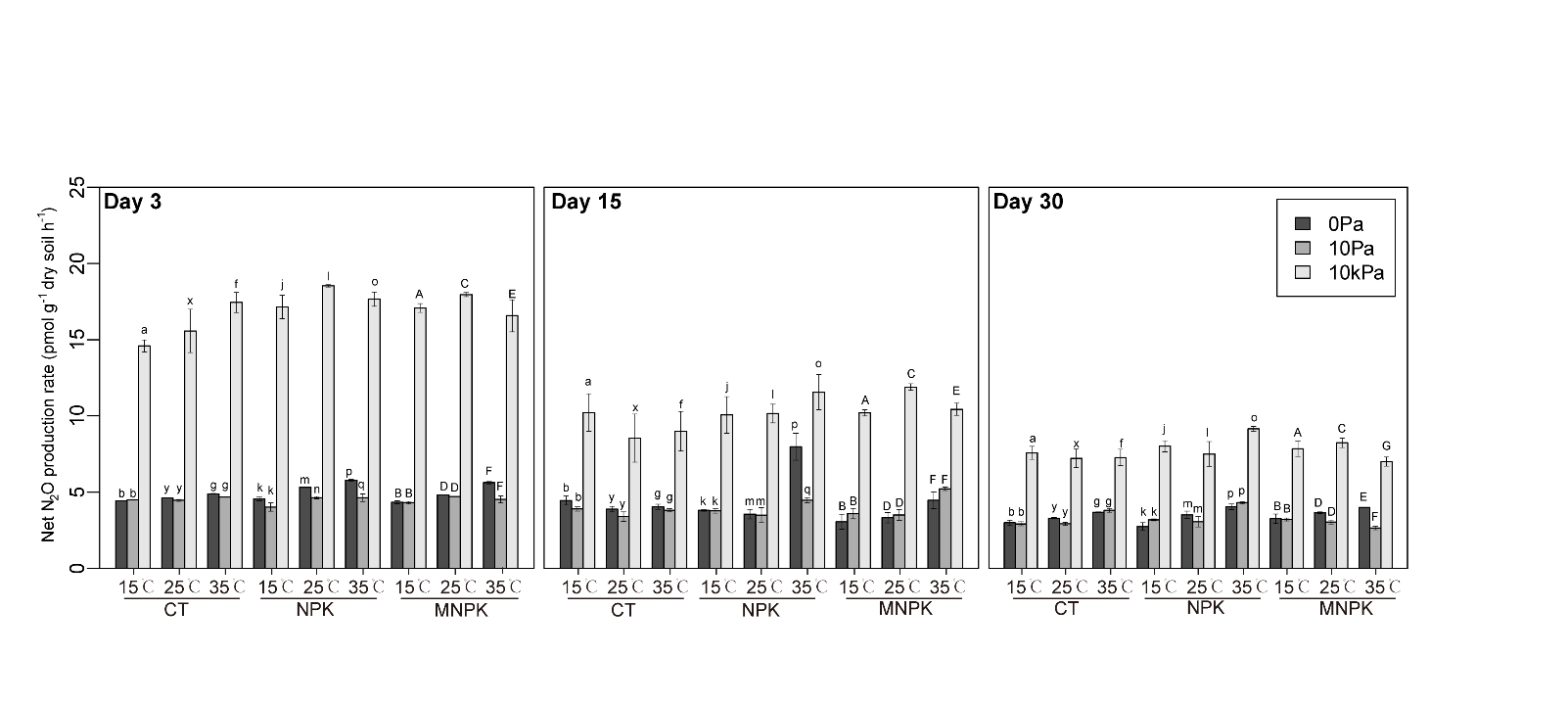
**Supplementary Figure 2.** The net N_2_O production rate of different C_2_H_2_ concentration treatments under different combination of fertilization and temperature treatments at Days 3,15, and 30. Pairwise comparison was conducted to estimate the differences of net N_2_O production rate among different C_2_H_2_ concentration treatments within combination of temperature and fertilization treatment. The different letters within same combination of temperature and fertilization treatment combination suggested significant difference between different C_2_H_2_ inhibition treatments at significance level *p* < 0.05. Note that, the different letters among different combination of temperature and fertilization treatments have no meaning.

**Supplementary Figure 3**. The bootstrapped results of gross N_2_O production (A), gross N_2_O consumption rate (B), and net N_2_O production rate (C) under different fertilization and temperature combinations at Days 3, 15, and 30. The bootstrap means (dots) and 95% CIs (bars) are shown (10 000 iterations).


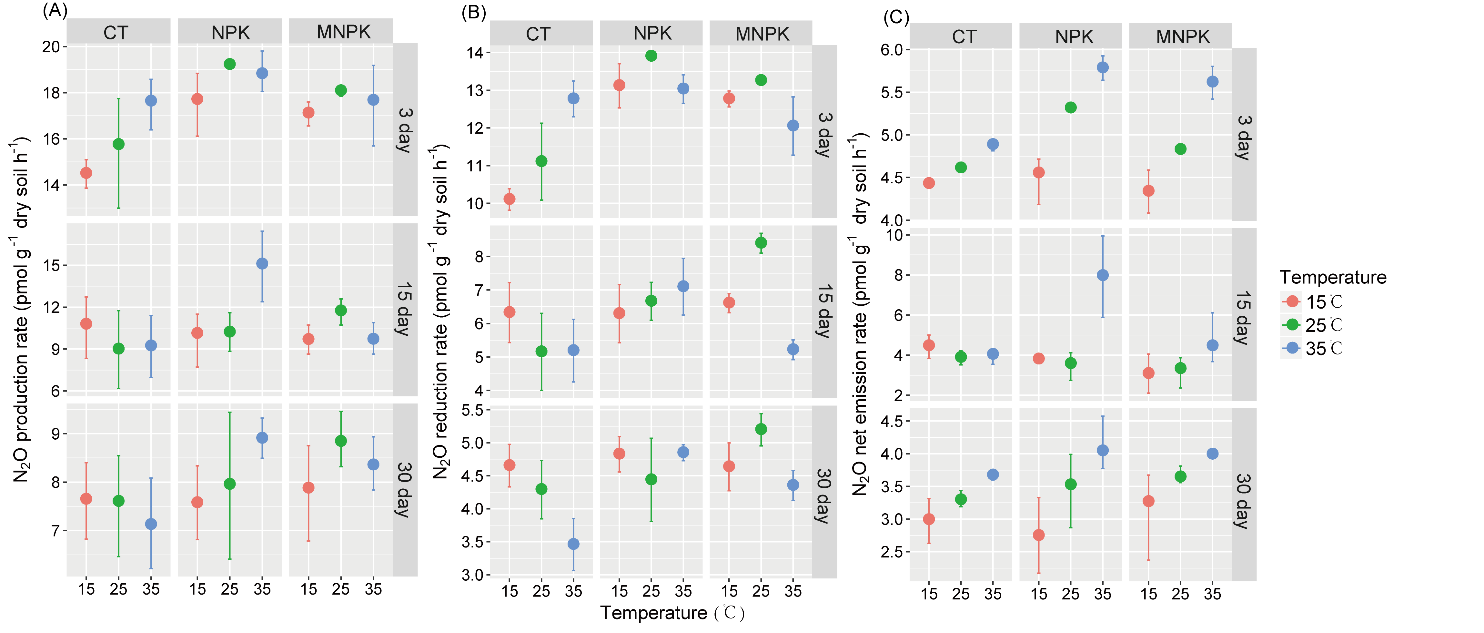


**Supplementary Figure 4.** The NH_4_^+^ (upper) and NO_3_^-^ (lower) content at Days 0, 3, 15, and 30. The means ± SE (n = 4) were shown.


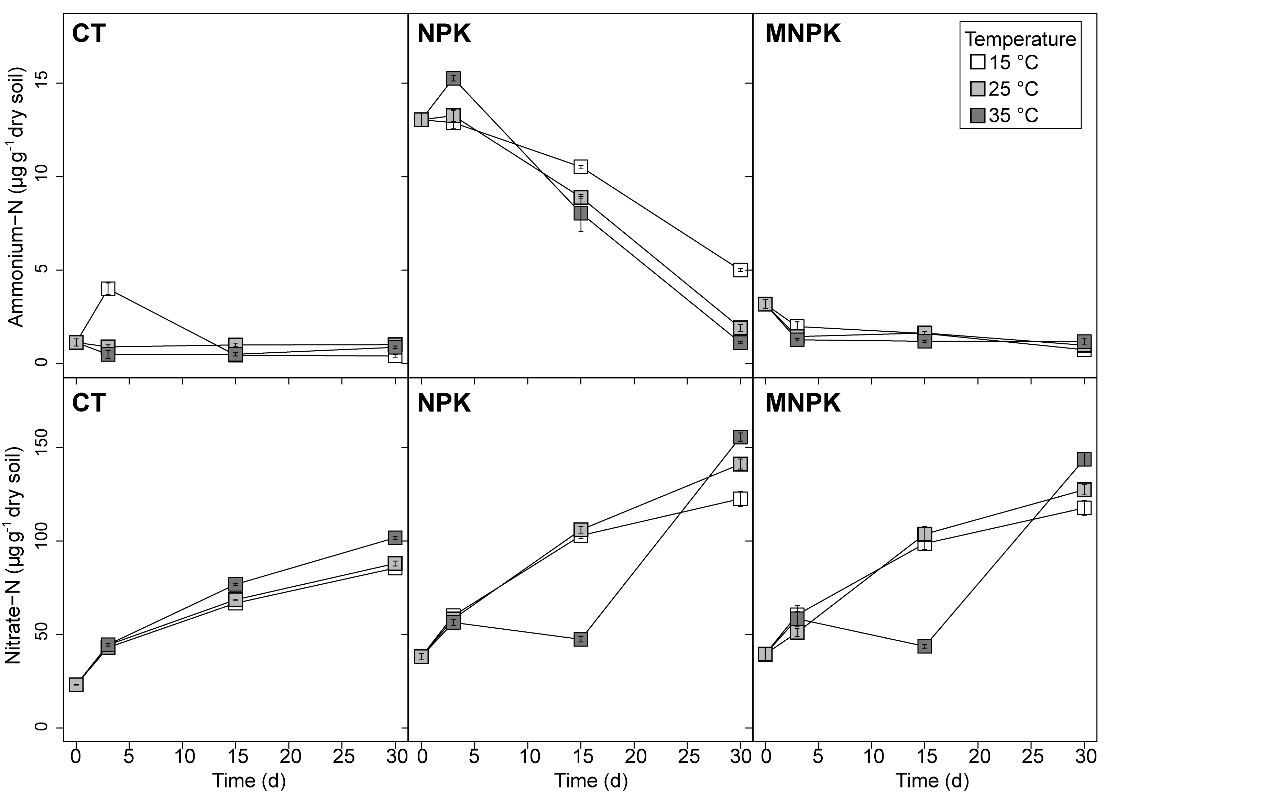


**Supplementary Figure 5.** The linear regression analysis for processes rates of interest. The dots represent the bootstrap means (1000 iterations), the bars stand for 95% CI. Only the linear regressions with the *p* < 0.05 were shown.


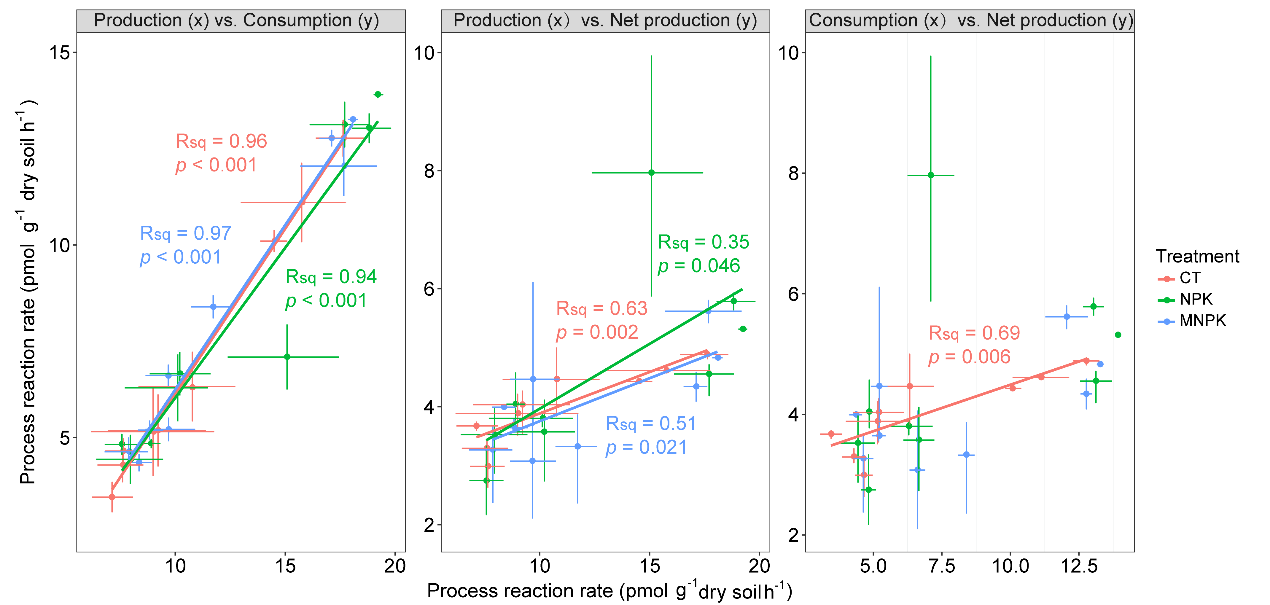


**Supplementary Figure 6.** The relative influence of covariates measured in predicting the net N_2_O emission.
